# Bilingual Language Control Flexibly Adapts to Cultural Context

**DOI:** 10.3389/fpsyg.2021.744289

**Published:** 2021-10-28

**Authors:** Cong Liu, Lu Li, Lu Jiao, Ruiming Wang

**Affiliations:** ^1^Department of Psychology, Normal College & School of Teacher Education, Qingdao University, Qingdao, China; ^2^Guangdong Provincial Key Laboratory of Mental Health and Cognitive Science, and Center for Studies of Psychological Application, School of Psychology, South China Normal University, Guangzhou, China; ^3^Bilingual Cognition and Development Lab, Center for Linguistics and Applied Linguistics, Guangdong University of Foreign Studies, Guangzhou, China

**Keywords:** language control, cultural context, bilinguals, switch cost, reversed language dominance effect

## Abstract

How does bilingual language control adapt to the cultural context? We address this question by looking at the pattern of switch cost and reversed language dominance effect, which are suggested to separately reflect reactive and proactive language control mechanisms, in the contexts with culturally-neutral pictures (i. e., baseline context) or culturally-biased pictures (i.e., congruent context where culture matched the language to be spoken or incongruent context where culture mismatched the language to be spoken). Results showed an asymmetric switch cost with larger costs for L2 in the congruent context as compared with the baseline and incongruent contexts, but the reversed language dominance effect was not changed across contexts, suggesting that cultural context plays a critical role in modulating reactive but not proactive language control. These findings reveal the dynamic nature of language control in bilinguals and have important implications for the current models of bilingual language control.

## Introduction

In a diverse cultural society, bilinguals accommodate the cultural background in their language acquisition (Kandhadai et al., 2014). For example, when learning the word “statue”, the Americans might always associate it with “Statue of Liberty”, but the Chinese might always associate it with “Statue of Confucius”. Hence, such special experience might lead to a critical role of the cultural context in multilingual communication.

During multilingual communication, bilinguals switch between their two languages they speak and utilize language control mechanisms to inhibit interference from the other language when aiming to speak in the intended language (Green, 1998; but see Blanco-Elorrieta and Caramazza, 2021; for a selection mechanism). Language control has been studied for years, and the recent evidence indicated that language control adapts flexibly depending on the language context (Timmer et al., 2019a,b) or the race of interlocutors' faces (Liu et al., 2019a). However, it remains unclear what is the influence of cultural context on bilingual language control? In the present study, we aim to examine whether language control in bilinguals flexibly adapts to socio-cultural contexts based on culturally-biased pictures.

### Language Control in Bilinguals

Previous studies have indicated that both languages in bilinguals are active simultaneously and interfere with each other (for a review, see Declerck and Philipp, 2015a). Thus, when aiming to speak in the intended language, a control process referred to as language control is implemented to minimize cross-language interference. The language switching task is typically used to examine the mechanism of language control (Meuter and Allport, 1999; Chang et al., 2016; Liu et al., 2019b), in which participants name items in either their first (L1) or second (L2) language depending on a cue. Within this task, two types of control processes were identified: reactive control as measured by the switch cost and proactive control as measured by the reversed language dominance effect (Liu et al., 2019a; Timmer et al., 2019b; Wu et al., 2017), the mixing cost (Ma et al., 2016; Timmer et al., 2019a) or the blocked language-order effect (Van Assche et al., 2013; Branzi et al., 2014; for a review, see Declerck, 2020). In the present study, we used the reversed language dominance effect to measure proactive control since most previous studies on language switching used this index (see a review, for Bobb and Wodniecka, 2013). During bilingual language switching, proactive language control was associated with the anticipation of speaking a target language and prevent potential cross-language interference before it occurs. Thus, it is more global (i.e., non-trial-specific) in nature. By contrast, the reactive control was associated with the resolution of interference from the non-target language after the activation of both languages (Ma et al., 2016). Therefore, it is more local (i.e., trial-by-trial) in nature.

As the index of reactive language control, the *switch cost* refers to the difference in response latencies or accuracy between repetition trials (i.e., repeat the same language as the previous one) and switch trials (i.e., switch from one language to another) in a language switching context. The magnitude of the switch cost seems to depend on language proficiency. It has been found that the switch cost is often asymmetrical (i.e., L2-L1 switch cost is larger than L1-L2 switch cost) for less-proficient unbalanced bilinguals (Meuter and Allport, 1999; Bobb and Wodniecka, 2013), and more symmetrical in high-proficient balanced bilinguals (Costa and Santesteban, 2004; Costa et al., 2006). The inhibitory control (IC) model proposed that, for unbalanced bilinguals, because the more dominant L1 requires more inhibition on L2-naming trials, it should take participants longer to switch into their L1 (Green, 1998).

As the index of proactive language control, the *reversed language dominance effect* refers to worse performance in L1 than that in L2 (i.e., slower naming or higher error rate in L1 than L2) in a language switching context (Christoffels et al., 2007, 2016; Wu et al., 2017; Liu et al., 2019a; Declerck et al., 2020). On the one hand, some studies (e.g., Christoffels et al., 2007; Gollan and Ferreira, 2009) indicated that the reversed language dominance effect comes about from consistently inhibiting L1 in a mixed language block. On the other hand, the other studies suggested that the constant increase in L2 activation throughout a mixed language block could account for this effect as well (e.g., Declerck et al., 2015).

### Language Switching Within the Cultural Context

The adaptive control hypothesis proposed that bilingual language control during language switching involves a dynamic set of adaptive changes depending on the context a bilingual resides in (Green and Abutalebi, 2013). In recent years, researchers have explored how linguistic context modulated the language control process (Declerck and Philipp, 2015b; Olson, 2015; Gollan and Goldrick, 2016; Timmer et al., 2019a,b). For instance, Timmer et al. (2019a,b) asked Dutch–English bilinguals to complete a language switching task in both a dominant L1 context with 83% pictures had to be named in Dutch and a non-dominant L2 context with 83% pictures had to be named in English. Finally, symmetric switch cost and reversed language dominance effect were observed in the L1 context, while an asymmetric switch cost with a larger cost for L2 and no reversed language dominance effect was found in the L2 context. This suggests that both local and global language control could be flexibly modulated by linguistic context.

While it seems that linguistic context shapes language control in bilinguals, only few studies have investigated how non-linguistic context shapes language control so far. As a non-linguistic factor, the culture cues such as faces and iconic images have been shown to play an important role in bilingual language processing (Li et al., 2013; Zhang et al., 2013; Berkes et al., 2018). However, there seems to be conflicting evidence regarding the effects of cultural factors on language control mechanisms (Roychoudhuri et al., 2016; Liu et al., 2019a). For instance, Roychoudhuri et al. (2016) asked Bengali-English bilinguals to name pictures with a background image (i.e., Bengali cultural images or neutral images) in both their L1 and L2. The authors found that switching cost and mixing cost were not modulated by the background image, although naming in English with iconic Bengali culture image was significantly slower than a neutral one. This indicated the workings of both reactive and proactive language control system was not modified by the non-linguistic cultural context. However, in one recent study, non-proficient bilinguals were instructed to name pictures with an Asian face or a Caucasian face, and the face matched (i.e., congruent context) or mismatched (i.e., incongruent context) the language of naming (Liu et al., 2019a). There was also a baseline context without the presence of faces. The results showed an asymmetric switch cost with larger costs for L2 in the congruent context as compared to the baseline and incongruent contexts, indicating the reactive language control was modulated by the non-linguistic contextual faces (see also Zheng et al., 2020). By contrast, the reversed language dominance effect kept the same across contexts, suggesting the proactive language control was not modulated by the non-linguistic contextual faces. Overall, it remains unclear whether or how the non-linguistic cultural context could modulate bilingual language control.

### The Present Study

In the present study, we examined what the effect of cultural context is on both reactive language control (i.e., switch cost) and proactive language control (i.e., reversed language dominance effect). Non-proficient Chinese–English bilinguals performed a language switching task in three contexts: (1) the baseline context, during which participants were naming a culturally-neutral picture in Chinese or English; (2) the congruent context, during which participants were naming a culturally-biased picture and the cultural bias conveyed in the picture was congruent with the language to be named (i.e., naming a Chinese-culture biased picture in Chinese or naming a western-culture biased picture in English); and (3) the incongruent context, during which the cultural bias conveyed in the picture was incongruent with the language to be named (i.e., naming a western-culture biased picture in Chinese or naming a Chinese-culture biased picture in English).

Given that both faces and iconic images are cultural cues, we expect the observed cultural context effect on language control induced by iconic images in the present study will be similar to the cultural context effect induced by faces in our previous study (Liu et al., 2019a). For reactive language control, we expect a different pattern of switch cost in the congruent context as compared to the baseline context. Specifically, the non-proficient Chinese-English bilinguals in the current study were more familiar with Chinese-culture biased pictures than western-culture biased pictures, as they were living in China since born. Moreover, they generally acquired their both L1 and L2 languages with the Chinese-culture biased pictures and always acquired their non-proficient L2 via the proficient L1 (see the Revised Hierarchical Model, for Kroll and Stewart, 1994), so the connection strengths between Chinese-culture biased pictures and their L1 labels should be stronger than the connections between western-culture biased pictures and their labels in L2. Thus, the culturally-biased pictures facilitated speech production when they matched the language (Jared et al., 2013), and the facilitation should be stronger for the dominant L1 than the non-dominant L2. Based on these, we predicted that switching back to the dominant language would be easier than switching back to the non-dominant language. Therefore, in the congruent context, an asymmetric switch cost with a larger switch cost toward L2 would be observed. By contrast, we expected the pattern of switch cost in the incongruent context would be the same as that in the baseline context. For proactive language control, on the other hand, we hypothesize that if cultural cues such as faces could not modulate the reversed dominance effect (Liu et al., 2019a), then these culturally-biased pictures should also show similar effects.

## Method

### Participants

Sixty-six non-English majors' students with normal or corrected-to-normal vision participated in this experiment. Seven participants were excluded from analysis due to having an accuracy lower than 75% (Liu et al., 2019a), leading to the final sample was 59 (41 females, age range 18–24 years). All participants signed informed consent before their participation and got paid after their participation. The experimental procedure was approved by the ethics committee of South China Normal University. We administered a self-rating language questionnaire in which participants assessed their Chinese and English language skills. These self-ratings were given on a 7-point scale with “1” being least proficient and “7” being most proficient. Paired-samples *t*-tests indicated that the proficiency ratings for all four language skills (i.e., listening, speaking, reading, and writing) in L1 were significantly higher than that in L2 (all *ts* > 10.176, all *ps* < 0.001, see Table 1).

**Table 1:** Means (and SDs) of language proficiency self-ratings and age of acquisition (AOA).

**Self-ratings**	**L1 (Chinese)**	**L2 (English)**
AOA		8.14 (2.23)
Listening	6.22 (0.72)	3.49 (1.29)
Speaking	6.08 (0.95)	3.32 (1.11)
Reading	6.10 (0.78)	4.34 (1.19)
Writing	5.75 (0.99)	3.78 (1.16)

### Materials

**Figure 1:**
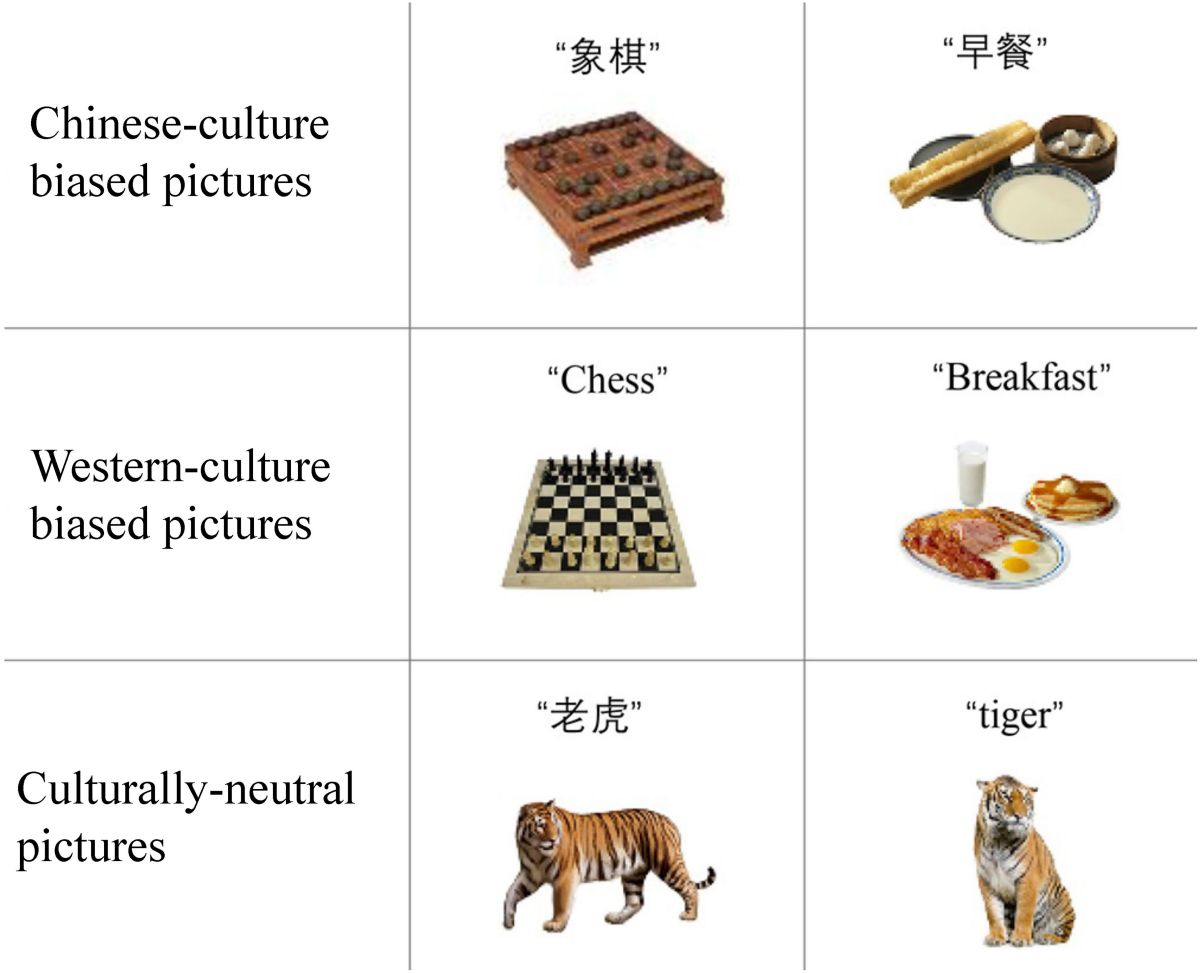
Sample pictures. There is a one-to-one correspondence between Chinese-biased pictures and western-biased pictures. For example, “早餐” in Chinese and “Breakfast” in English indicate the same object. To match the biased pictures, there are two pictures indicating the same object for culturally-neutral pictures as well.

One hundred twenty four pictures (31 Chinese-culture biased pictures; 31 Western-culture biased pictures, and 62 culturally-neutral pictures) were used in the present study (see Figure 1 for sample pictures; also see Appendix for all culturally-biased pictures), four of which were used as filler trials. These pictures were selected in the following steps: (1) we preliminarily selected about 70 pairs of culturally-biased pictures and 40 pairs of neutral pictures by searching the internet; (2) then edited these pictures into the same format with Photoshop; (3) 22 students were recruited to rate the pictures for their familiarity, typicality, visual complexity, and relevance to Chinese or western culture on a seven-point Likert scale. For the familiarity, typicality, and visual complexity, 1 indicating “not at all” and 7 indicating “very”. For cultural bias, 1 indicating “relevance to western culture” and 7 indicating “relevance to Chinese culture”. They were also asked to type in the most appropriate Chinese and English names of each picture. (4) Pictures with the low naming agreement were excluded and we finally selected the pictures based on their self-rated attributes. See Table 2 for the mean ratings for the attributes of pictures used in the present study.

**Table 2:** Attributes for experimental materials.

	**Chinese-culture**	**Western-culture**	**Culturally-neutral**
	**biased pictures**	**biased pictures**	**pictures**
Familiarity	6.30(0.25)	6.26(0.35)	6.46(0.32)
Typicality	6.39(0.27)	6.28(0.37)	6.40(0.37)
Cultural bias	1.68(0.37)	5.79(0.51)	4.11(0.40)
Visual Complexity	2.52(0.86)	2.62(0.69)	2.43(0.54)
Number of characters for L1 labels	1.90(0.30)	1.90(0.30)	1.87(0.43)
Number of syllables for L2 labels	5.77(1.36)	5.77(1.36)	5.45(1.18)

One-way ANOVA was conducted for each self-rated attribute to compare the differences across three types of pictures. The results showed that there were no significant differences between the three picture types for familiarity, typicality, visual complexity, and number of characters for L1/L2 labels (*ps* > 0.05). However, there was a significant difference for cultural bias, and the *post-hoc* tests revealed a significant difference among the three picture types respectively (*ps* < 0.05) (see Table 2).

### Task and Procedure

**Figure 2:**
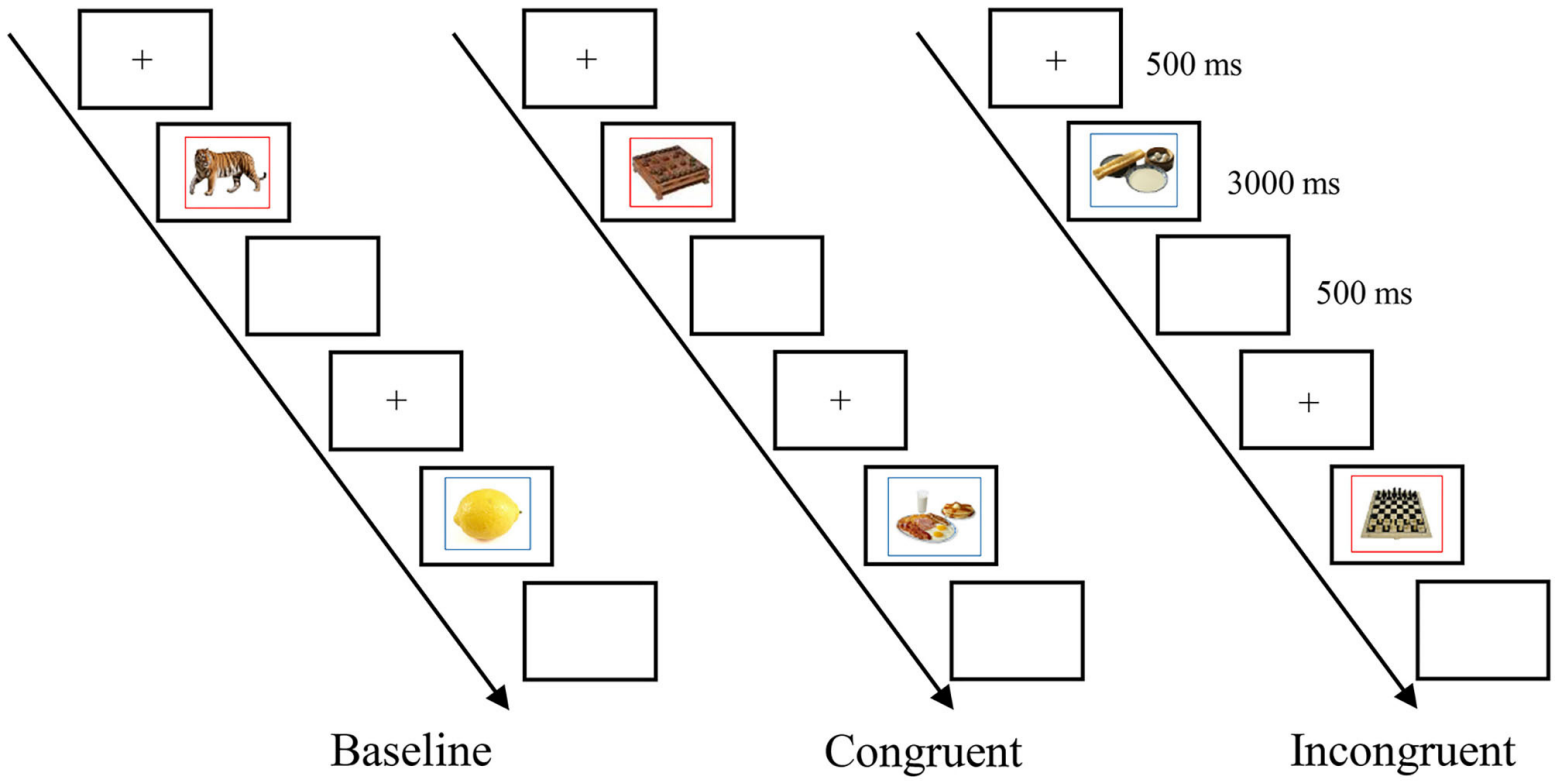
The trial procedure for three contexts.

The current experiment used a cued picture naming task. Participants familiarized themselves with the names in both L1 and L2 for the pictures before the experiment. The familiarization was self-paced, during which the pictures with their L1 or L2 names were presented one by one. Half of the participants first familiarized the L1 names of the pictures, while the other half first familiarized the L2 names. As depicted in Figure 2, the procedure was the following for each trial: A fixation point was presented on the computer screen for 500 ms; a picture of an object was presented together with the colored frame and remained on the screen until the participant name the picture or a maximum duration of 3,000 ms; and a blank screen appeared for 500 ms. Participants were instructed to name the picture in Chinese for the red frame and in English for the blue frame.

There were three blocks (i.e., a baseline block, a congruent block, and an incongruent block) in the cued picture naming task, and the block order was counterbalanced across participants. In the baseline block, the participants were naming a culturally-neutral picture in Chinese or English. In the congruent block, the participants were naming a Chinese-culture biased picture in Chinese or naming a western-culture biased picture in English. In the incongruent block, the participants were naming a western-culture biased picture in Chinese or naming a Chinese-culture biased picture in English. Each block included 61 trials, with the first trial being the filler trial, and there will be an equal number of trials per trial type (i.e., 15 trials for L1-L1 repeat, L2-L1 switch, L2-L2 repeat, and L1-L2 switch). There was a practice block with 12 trials before the formal experimental blocks. To check the accuracy of verbal responses made by the participants, an “EV Capture” screen recording software was used to record the whole experimental process.

### Analysis

For RT analyses, we excluded the first trial of each block, the error trials (including trials named incorrectly, trials named in an incorrect language, or trials without any response), as well as the trials following an error trial. Trials with RTs that beyond Mean ± 2.5 SD were excluded as well (Liu et al., 2019a). RT data were analyzed in R (version 3.5.0) using linear mixed-effects models. We always firstly built a full model including all fixed effects and random effects. Then, by removing the random effects which did not improve model fit (*ps* > 0.1) (Barr, 2013), we eventually fit a model, with RTs as the dependent variable and Contexts (baseline vs. congruent vs. incongruent), Language (L1 vs. L2), Transition (repetition vs. switch), and their interactions as fixed effects. Besides, the model included by-participant and by-item random intercepts, by-participant random slopes for contexts. In this model, the factor Language was coded as −0.5 for L1 and as 0.5 for L2, and factor Transition was coded as −0.5 for repetition and 0.5 for switch. This mean-centered contrast coding method yields results directly analogous to that obtained from an ANOVA. The factor context was coded with dummy coding so that the baseline context was taken as the reference level to which all other levels are compared.

Similarly, the logistic mixed-effects model was fitted on accuracy data, with the same fixed- and random-effects structure as in the linear mixed-effects model on RT.

## Results

As shown in **Table 4**, the model on RT data showed a significant effect of Language (*t* = −3.23, *p* = 0.002, *d* = 0.23), indicating slower naming in L1 than L2 (i.e., reversed dominance effect) in baseline context. Moreover, the Language did not interact with both Congruent and Incongruent contexts (*p*s > 0.05), suggesting the same *reversed language dominance effect* across contexts. Overall, this finding suggested that the cultural contexts could not modulate the reversed language dominance effect.

**Figure 3:**
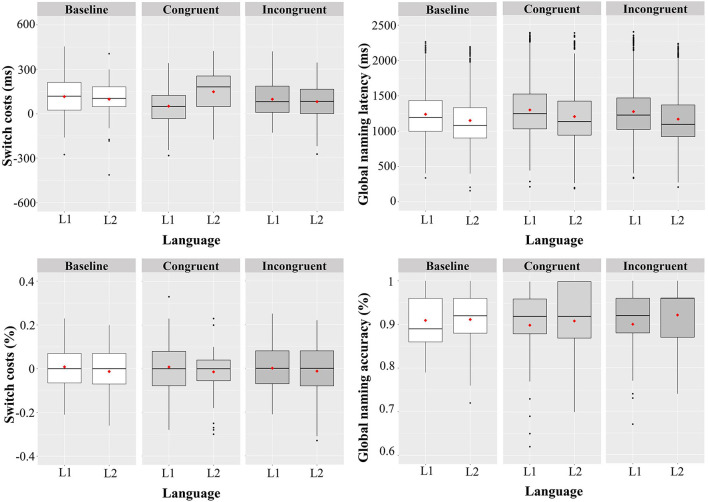
Boxplots showing the language switch cost **(left)** and reversed dominant effect **(right)** in RTs **(top panel)** and accuracy **(below panel)** across cultural contexts. The boxplot shows the interquartile range with the black dots representing the outliers falling outside the 1.5*interquartile range. The median is indicated by the horizontal black line and the centers of the red quadrangle indicated the means.

The significant effect of Transition (*t* = 8.55, *p* < 0.001, *d* = 0.28) suggested that the switching trials are significantly slower than repetition trials (i.e., switch cost) in baseline context. Further, the Transition did not interact with both Congruent and Incongruent contexts (*p*s > 0.05), suggesting the same *switch cost* across contexts. The two-way interaction between Language and Transition was non-significant (*t* = −0.35, *p* = 0.727), indicating a *symmetrical switch cost* in the baseline context. Crucially, the significant three-way interaction between Congruent, Language and Transition (*t* = 2.43, *p* = 0.015, *d* = 0.23) indicating a different switch cost pattern in the congruent context as compared to the baseline context and the non-significant three-way interaction between Incongruent, Language and Transition (*t* = 0.04, *p* = 0.972) indicating a similar switch cost pattern in incongruent context as compared to the baseline context. To further reveal the exact patterns of switch cost in both congruent and incongruent contexts, separate submodels were conducted. In the congruent context, we found a significant interaction between Language and Transition (*t* = 3.12, *p* = 0.002, *d* = 0.21), indicating an *asymmetrical switch cost* with larger costs for L2. In contrast, we found a non-significant interaction between Language and Transition in the incongruent context (*t* = 0.15, *p* = 0.883), indicating a *symmetrical switch cost*. Taken together, these findings suggest that the cultural contexts could modulate the switch cost (see Tables 3, 4, Figure 3).

**Table 3:** Mean RTs and Accuracy for all three contexts (standard deviations in parentheses).

	**Baseline**		**Congruent**		**Incongruent**	
	**L1**	**L2**	**L1**	**L2**	**L1**	**L2**
*RT*						
Repetition	1184(320)	1100(336)	1270(389)	1129(332)	1216(342)	1121(350)
Switch	1292(334)	1201(331)	1320(352)	1279(373)	1313(354)	1197(346)
*ACC*						
Repetition	0.91(0.07)	0.92(0.07)	0.89(0.14)	0.91(0.10)	0.90(0.10)	0.93(0.08)
Switch	0.91(0.08)	0.91(0.10)	0.90 (0.10)	0.90(0.11)	0.90(0.09)	0.91(0.10)

**Table 4:** Mixed-effects model for RTs.

**Fixed effects**	**Estimate**	***SE***	***t*-value**	***p*-value**
Intercept	1,199.59	22.78	52.65	** <0.001**
Context: Congruent	61.52	22.49	2.74	**0.007**
Context: Incongruent	22.90	21.18	1.08	0.282
Language	−83.78	25.91	−3.23	**0.002**
Transition	100.63	11.77	8.55	** <0.001**
Congruent × Language	−8.76	37.45	−0.23	0.815
Incongruent × Language	−23.42	37.41	−0.63	0.533
Congruent × Transition	−1.55	16.85	−0.09	0.927
Incongruent × Transition	−0.53	16.84	−0.03	0.975
Language × Transition	−8.23	23.54	−0.35	0.727
Congruent × Language × Transition	82.07	33.72	2.43	**0.015**
Incongruent × Language × Transition	1.18	33.67	0.04	0.972

*Significant p-values are highlighted in bold*.

As can be seen in Table 5, the model on accuracy data showed no significant effects for all factors and their interactions (*ps* > 0.05, see Figure 3).

**Table 5:** Mixed-effects model for ACCs.

**Fixed effects**	**Estimate**	***SE***	***z*-value**	***p*-value**
Intercept	2.46	0.11	23.38	** <0.001**
Context: Congruent	0.04	0.15	0.27	0.791
Context: Incongruent	0.01	0.13	0.05	0.963
Language	0.02	0.14	0.15	0.879
Transition	−0.04	0.13	−0.29	0.774
Congruent × Language	0.14	0.20	0.69	0.492
Incongruent × Language	0.23	0.20	1.17	0.243
Congruent × Transition	0.03	0.18	0.16	0.871
Incongruent × Transition	−0.05	0.18	−0.26	0.796
Language × Transition	−0.26	0.25	−1.03	0.304
Congruent × Language × Transition	−0.06	0.36	−0.18	0.860
Incongruent × Language × Transition	0.07	0.36	0.20	0.843

*Significant p-values are highlighted in bold*.

## Discussion

This study investigated the issue of whether reactive and proactive language control would be influenced by the cultural context. Non-proficient Chinese–English bilinguals performed a cued language switching task in three different contexts: congruent (i.e., naming a Chinese-culture biased picture in Chinese or naming a western-culture biased picture in English), incongruent (i.e., naming a western-culture biased picture in Chinese or naming a Chinese-culture biased picture in English), and baseline (i.e., naming a culturally-neutral picture) contexts. The results showed that cultural context could affect reactive language control when the culturally-biased pictures matched the language, but did not affect proactive language control.

First, a unique pattern of language switch cost was observed in the congruent context as compared to the baseline context. Contrary to the typical asymmetry where there is a larger switch cost for the L1 than the L2, we found an asymmetrical switch cost with a larger cost for the L2 than the L1 in the congruent context, whereas a symmetric switch cost was observed in the baseline context. Regarding this result, the most likely explanation is that the activation threshold to access the lexical representation of both languages in bilinguals is altered by the culturally-biased pictures. The classical IC model indicated that, for unbalanced bilinguals, L1 representations need to be inhibited to a larger extent than L2 representations. During bilingual language production, the L1 with a higher activation level is inhibited largely when speaking in the L2. Then, when switching back to the L1, this language needs to be re-activated, making it harder to access L1 words (Green, 1998). Previous research has indicated that cultural factors such as faces with socio-cultural identity (Li et al., 2013) and culturally-biased pictures (Jared et al., 2013) facilitated speech production when they matched the language to be spoken. Moreover, as Chinese-English bilinguals were more familiar with the cultural backgrounds behind Chinese-culture biased pictures than western-culture biased pictures, and generally acquired their both languages with the Chinese-culture biased pictures, the connection strengths between Chinese-culture biased pictures and their labels in L1 are stronger than the connections between western-culture biased pictures and their labels in L2, finally leading to this facilitation was stronger for the L1 than the L2. In the congruent context, when the unbalanced Chinese-English bilinguals name a picture on a switch trial toward Chinese, the lexical representations for Chinese are primed by the Chinese-biased picture, making it easier to re-activate the strongly suppressed Chinese. Thus, this reduces the switch cost for L1 compared with L2.

Apart from the IC model, another hypothesis of language control (i.e., Persisting Activation Hypothesis; see Philipp et al., 2007) could help account for the observed asymmetrical switch cost in the congruent context as well. The Persisting Activation Hypothesis proposed that, in switch trials, the persisting activation of the naming language in the present trial interferes with the new naming language in the next trial, leading to switch cost. Besides, an asymmetrical switch cost with a larger switch cost in L1 would be expected as the non-dominant L2 needs to be activated to a stronger level to be produced. However, in the present study, the different facilitation effects of culturally-biased pictures would alter the directions of asymmetry. Specifically, as Chinese-English bilinguals are more familiarized with the cultural backgrounds behind Chinese-culture biased pictures, the facilitation of Chinese-culture biased pictures for L1 is stronger than the facilitation of Western culturally-biased pictures for L2 when the culturally-biased pictures matched the language to be spoken. Consequently, the dominant L1 was activated to a stronger level as compared to the non-dominant L2, which eventually resulting in a larger switch cost in L2 than L1. It should be noted that, in the congruent context, switch cost in L1 is relatively small (50 ms), and switch cost in L2 is relatively large (150 ms) as compared to the switch cost in the baseline context (108 ms in L1 and 97 ms in L2). We speculated that this smaller switch cost in L1 might arise from the facilitating effect of familiar culture backgrounds behind the Chinese-culture biased pictures, while this larger switch cost in L2 might arise from the high cognitive demands on unfamiliar foreign culture backgrounds behind the English-biased pictures.

Second, a symmetrical switch cost pattern was observed in the incongruent context, which was the same as in the baseline context. This is consistent with one recent study which revealed that the incongruent contextual faces could not affect the switch cost pattern (Liu et al., 2019a). Socio-cultural faces and culturally-biased pictures are all cultural factors. When both are incongruent with the language to be spoken, there was a general interference effect for lexical access or language production (see Zhang et al., 2013; Roychoudhuri et al., 2016; Berkes et al., 2018). However, the results in the current study and Liu et al. (2019a) suggested that such incongruent cultural context with faces or culturally-biased pictures could not affect the reactive language control.

In addition, the reversed language dominance effect keeps the same across the different cultural contexts, suggesting that proactive language control was not adjusted based on cultural context. It seems that the influence of the cultural context on the relative activation of each language was only at a trial-by-trial level instead of a global level. These are in contrast to the previous findings which indicated that linguistic language context could shape both reactive and proactive language control (Wu et al., 2017; Timmer et al., 2019a,b). Taken together, these findings suggested that linguistic and non-linguistic contexts affect language control in different ways.

Overall, the present study showed non-linguistic cultural context affected reactive language control but not proactive language control, which was consistent with one previous study revealing that non-linguistic contextual faces also affect reactive language control but not proactive language control (Liu et al., 2019a). However, by contrast, Roychoudhuri et al. (2016) found no effect of non-linguistic cultural context on both reactive and proactive language control. For such different findings, we argue that it might arise from the difference in the manipulation of the cultural contexts. In Roychoudhuri et al. (2016), the participants switched between their both languages in the background only with an iconic L1 (i.e., Bengali) culture picture, meaning that there were both congruent and incongruent trials during language switching. However, the participants in the current study switched between their languages with both iconic L1 and L2 culture pictures in congruent and incongruent cultural contexts, separately. Thus, the difference in cultural contexts might eventually induce different contextual effects. To better examine how non-linguistic cultural context modulates language control, researchers should conduct further investigations by creating more naturalistic cultural contexts with virtual reality (for a review, see Peeters, 2019).

The findings in the present study supported and expanded the adaptive control hypothesis (Green and Abutalebi, 2013), which proposed that linguistic interactional contexts (i.e., single-language context, dual-language context, and dense code-switching context) place a different level of demand on the cognitive systems and adaptively alter their language control (see Timmer et al., 2019a,b; Liu et al., 2020, 2021) and cognitive control processes (see Lai and O'Brien, 2020; Rafeekh and Mishra, 2021). The present study examined how non-linguistic cultural context shapes language control in bilinguals and found different patterns of language switch cost across contexts, suggesting that non-linguistic interactional contexts could shape the reactive language control processing. This finding aligns with the adaptive control hypothesis which proposes the adaptive nature of language control. Combined with the findings in one recent study which found that non-linguistic contextual faces modulated language control (Liu et al., 2019a), we proposed that the non-linguistic contexts should be introduced into the adaptive control hypothesis as well. Overall, not only linguistic contexts but also non-linguistic contexts play critical roles in shaping bilingual language control. In the future, more studies should be conducted to further investigate how language control changes depending on various processing contexts (Green and Abutalebi, 2013). This would enable us to better understand the adaptive bilingual language control.

## Conclusion

Taken together, this study showed different patterns of language switch cost but similar reversed language dominant effects across contexts with different culturally-biased pictures. These findings indicate that the cultural context only plays an important role in modulating reactive language control but not proactive language control in bilinguals.

## Data Availability Statement

The raw data supporting the conclusions of this article will be made available by the authors, without undue reservation.

## Ethics Statement

The studies involving human participants were reviewed and approved by the Research Ethics Committee of South China Normal University. The patients/participants provided their written informed consent to participate in this study.

## Author Contributions

CL and RW contributed to the conception and design of the study. CL and LL collected the data. LL and CL performed the statistical analysis. CL wrote the first draft of the manuscript. All authors contributed to manuscript revision, read, and approved the submitted version.

## Funding

This research was supported by Qingdao Planning Office of Philosophy and Social Science (QDSKL2001098) and a research grant (No. BCD202106) from the Bilingual Cognition and Development Lab, Center for Linguistics and Applied Linguistics, Guangdong University of Foreign Studies.

## Conflict of Interest

The authors declare that the research was conducted in the absence of any commercial or financial relationships that could be construed as a potential conflict of interest.

## Publisher's Note

All claims expressed in this article are solely those of the authors and do not necessarily represent those of their affiliated organizations, or those of the publisher, the editors and the reviewers. Any product that may be evaluated in this article, or claim that may be made by its manufacturer, is not guaranteed or endorsed by the publisher.
