# Supplementary material for: Bilingual Language Control Flexibly Adapts to Cultural Context
Source: Front Psychol. 2021 Oct 28;12:744289. doi: 10.3389/fpsyg.2021.744289 (PMC8581538; doi:10.3389/fpsyg.2021.744289)
Supplement: Supplementary file 1 [file Image_1.pdf]

Appendix: culturally-biased pictures

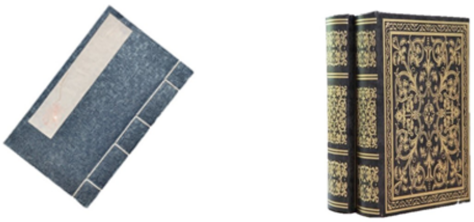

Book

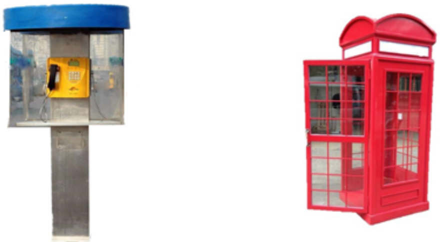

Booth

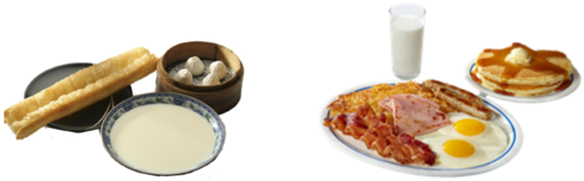

Breakfast

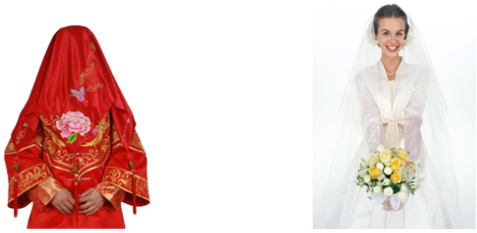

Bride

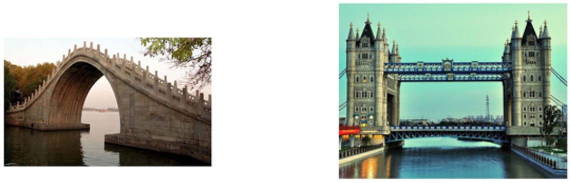

Bridge

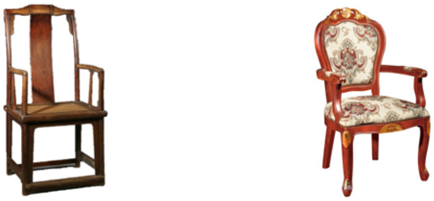

Chair

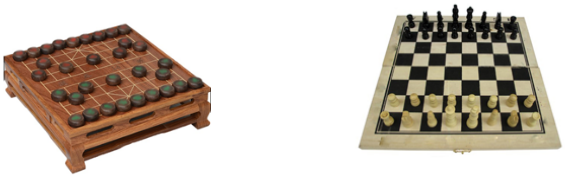

Chess

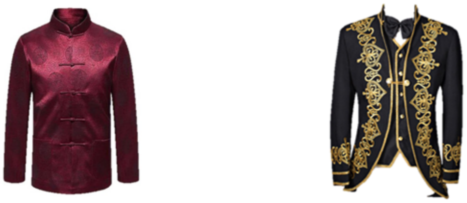

Coat

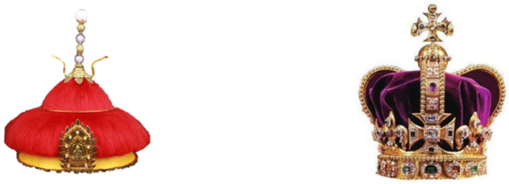

Crown

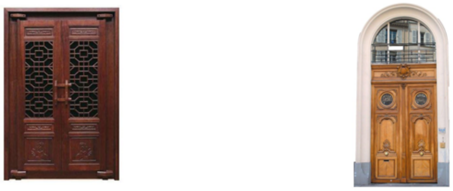

Door

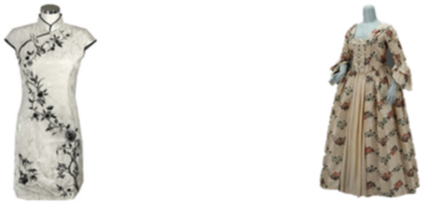

Dress

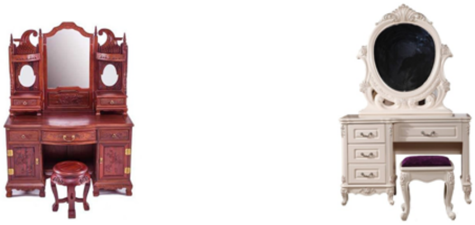

Dresser

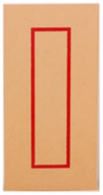 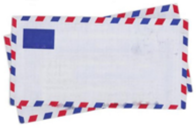

Envelope

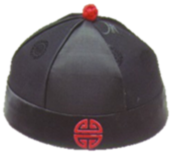 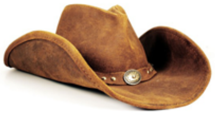

Hat

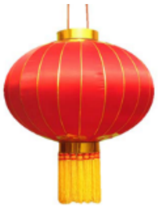 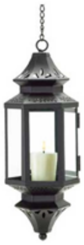

Lantern

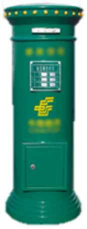 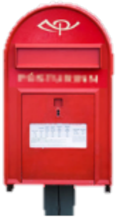

Mailbox

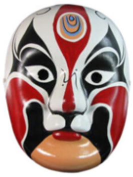 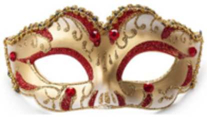

Mask

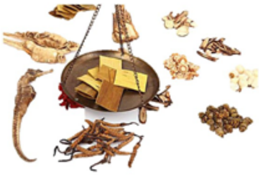 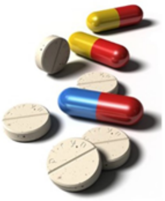

Medicine

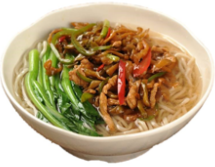 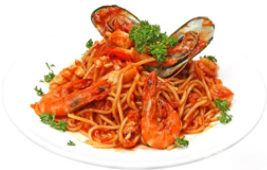

Noodles

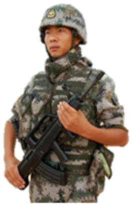 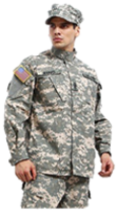

Soldier

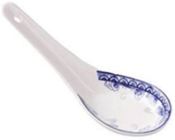 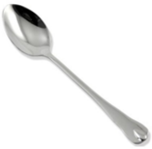

Spoon

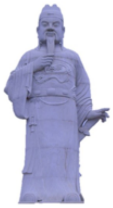 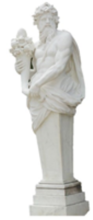

Statue

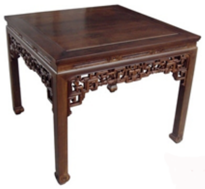 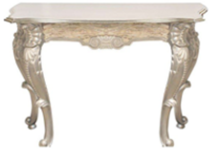

Table

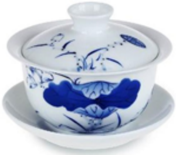 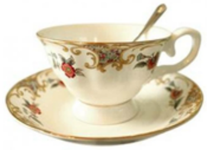

Teacup

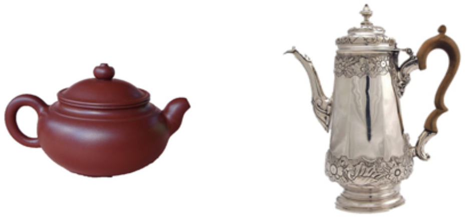

Teapot

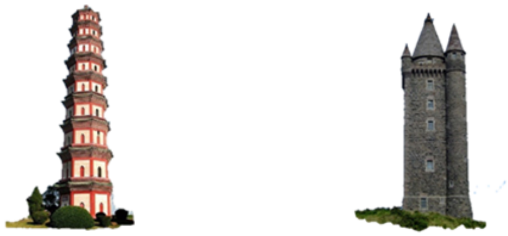

Tower

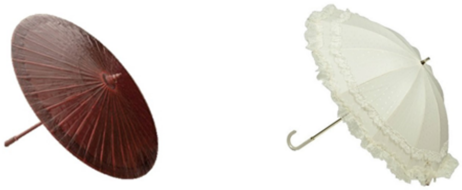

Umbrella

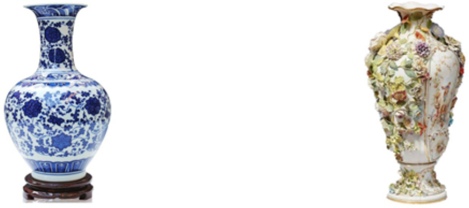

Vase

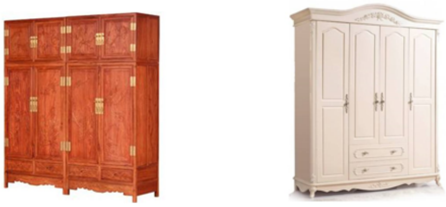

Wardrobe

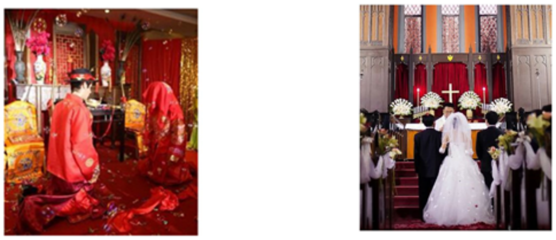

Wedding

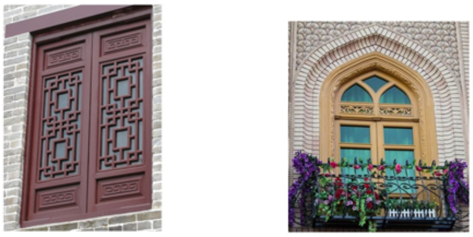

Window
